# Dynamic analysis of lung metastasis by mouse osteosarcoma LM8: VEGF is a candidate for anti-metastasis therapy

**DOI:** 10.1007/s10585-012-9543-8

**Published:** 2012-10-18

**Authors:** Takaaki Tanaka, Yoshihiro Yui, Norifumi Naka, Toru Wakamatsu, Kiyoko Yoshioka, Nobuhito Araki, Hideki Yoshikawa, Kazuyuki Itoh

**Affiliations:** 1 Department of Biology, Osaka Medical Center of Cancer and Cardiovascular Diseases, 1-3-3 Nakamichi, Higashinari-ku, Osaka, 537-8511 Japan; 2 Department of Orthopaedic Surgery, Osaka University Graduate School of Medicine, 2-2 Yamadaoka, Suita, Osaka, Japan; 3 Muscloskeletal Oncology Service, Osaka Medical Center of Cancer and Cardiovascular Diseases, 1-3-3 Nakamichi, Higashinari-ku, Osaka, 537-8511 Japan; 4 Department of Surgery, University of California, San Francisco, 513 Parnassus Ave, San Francisco, CA USA

**Keywords:** Osteosarcoma, Metastasis, Circulating tumor cells, Vascular endothelial growth factor, Transendothelial migration, Pazopanib

## Abstract

Osteosarcoma (OS) is the most common malignant bone tumor and the prognosis depends on pulmonary metastases, which arise from multi-step progression of malignant tumors. We herein aimed to clarify the critical step of pulmonary metastasis using the syngeneic mouse spontaneous highly metastatic OS LM8 and parental Dunn cell lines, to identify new candidate molecules to suppress pulmonary metastasis. We first investigated the chronological detection of circulating tumor cells (CTCs) from mice with either cell line. LM8 CTCs appeared faster, at a higher rate and with a greater number compared to Dunn CTCs. Cultured cells from CTCs of LM8 showed higher proliferative ability than cells from the primary site in suspension culture, which mimicked the environment of the bloodstream for CTCs. The proliferative ability of LM8 cells was also higher than that of Dunn cells in 3D collagen culture with low stiffness (−150 Pa; close to conditions in the lung). We next focused on the extravasation step. LM8 showed higher migration ability compared to Dunn with transendothelial migration assay. We also found a disruption in endothelial barrier function throughout co-culture with LM8 using time-lapse imaging. In addition, LM8 secreted high levels of vascular endothelial growth factor (VEGF), while VEGF signal inhibition with a small molecule tyrosine kinase inhibitor (pazopanib) decreased disruption of the vascular barrier and transendothelial migration of LM8. Finally, daily oral administration of pazopanib reduced the rate and size of pulmonary metastasis in vivo. Collectively, these results show anti-VEGF therapy as a candidate for pulmonary metastasis of OS.

**Electronic supplementary material:**

The online version of this article (doi:10.1007/s10585-012-9543-8) contains supplementary material, which is available to authorized users.

## Introduction

Osteosarcoma (OS) is the most common malignant bone tumor in adolescents and childhood. Despite combination treatment with wide resection and chemotherapy, lung metastases occur in ~40–50 % of OS patients [1, 2], resulting in poor prognosis [2]. Elucidation of the underlying mechanisms and new targets for the treatment of lung metastasis are strongly needed to improve prognoses for OS patients. The metastatic cascade in malignant tumor involves a multi-step progression: detachment and local invasion at the primary site, entry into the circulation (intravasation), survival in the bloodstream, migration though the endothelium (extravasation), and colonization of target organs [3–7]. The details of the metastatic process remain mysterious due to difficulties in studying cell behavior at sufficiently high spatial and temporal resolution in vivo [5]. We have performed detailed dynamic analysis of each step of the metastatic cascade using the LM8 mouse spontaneous highly metastatic OS cell line [8] and the parental Dunn line [9]. This syngeneic metastatic model offers the benefit of allowing us to investigate the biological features of metastasis through comparison of results for LM8 and Dunn cells against a consistent genetic background. We have already reported that LM8 cells show high secretion vascular endothelial growth factor (VEGF) [8], a fibroblastic morphology with abundant filopodia and invasive properties [8, 10] and migration ability for approximately two times in migration assays using a Boyden chamber [10, 11], which showed activated Cdc42 and autophosphorylation of focal adhesion kinase compared to Dunn cells [10].

The presence of circulating tumor cells (CTCs) in the bloodstream fits very well with this cascade, and is involved in intravasation, survival in circulation and extravasation steps, consequently detection of CTCs has long been considered as a possible tool for assessing the aggressiveness of tumors and subsequent development in distant organs [7, 12, 13]. VEGF as a key protein involved in the angiogenic switch in tumors [14], is also known as a vascular permeability factor and has been reported to decrease biological barrier function, promoting vascular permeability and extravasation via VEGF–VEGFR interactions [15, 16]. Kaya et al. [17, 18] reported serum VEGF levels as a predictor of lung metastasis and poor prognosis in patients with OS. This study aimed to clarify critical steps toward pulmonary metastasis using LM8 and parental Dunn, in order to seek new candidate molecules for suppressing the development of lung metastasis.

## Materials and methods

### Reagents

Pazopanib was kindly gifted from GlaxoSmithKline (London, UK). Recombinant murine VEGF165 was purchased from PeproTech (Rocky Hill, NJ). FITC-dextran was purchased from TdB Consultancy AB (Uppsala, Sweden). The collagen gel culture kit was purchased from Nitta Gelatin (Osaka, Japan). The Mouse VEGF Quantikine enzyme-linked immunosorbent assay kit was purchased from R&D Systems (Minneapolis, MN). CellTracker^TM^ Red CMTPX was purchased from Invitrogen (Carlsbad, CA).

### Cells

The LM8 highly metastatic OS cell line was derived from the Dunn OS cell line through eight repeated cycles of the procedure described by Poste and Fidler [19]. Mouse aortic endothelial cells (mAEC) were purchased from Angio-Proteomie (Boston, MA). LM8 and Dunn cells were maintained in Dulbecco’s modified Eagle’s medium (DMEM) (Invitrogen, Carlsbad, CA) supplemented with 10 % fetal bovine serum (FBS), and mAEC were maintained in DMEM supplemented with 20 % FBS. Cells were cultured at 37 °C in a fully humidified incubator under 5 % CO_2_.

### CTCs culture

Forty-microliter peripheral blood samples were collected from the tail vein and 1 μl of EDTA (0.5 μmol) added. These samples were maintained in DMEM supplemented with 10 % FBS and penicillin (100 U/ml)-streptomycin (100 μg/ml). All cells were cultured at 37 °C in a fully humidified incubator under 5 % CO_2_.

### Suspension culture

Poly-hydroxyethyl methacrylate (poly-HEMA) (Sigma, St. Louis, MO) was solubilized in 95 % methanol (30 mg/ml). To prepare poly-HEMA-coated dishes, 25-μl aliquots of poly-HEMA solution were placed onto 96-well dishes and dried in a tissue culture hood. Five thousand trypsinized LM8 or Dunn cells per 100 μl of medium were plated onto 96-well poly-HEMA-coated dishes and cultured for 24–48 h.

### Cell proliferation assay

Cell proliferation was measured using CellTiter-Glo Luminescent Cell Viability Assay (Promega, Madison, WI) according to the protocols provided by the manufacturer.

### Measurement of caspase-3/7 activity

Caspase-3/7 activation was measured using the Caspase-Glo 3/7 Luminescence Assay (Promega) according to the protocol from the manufacturer.

### Three dimensional culture of LM8 and Dunn

To assess cell morphology and proliferation in 3D collagen matrix, LM8 and Dunn cells were cultured using a 3D collagen cell culture system (Nitta Gelatin, Osaka, Japan). Briefly, collagen gel solution was prepared according to the instructions from the manufacturer. Collagen gel concentration at the bottom layer was 2.4 mg/ml, as while 1.2 or 2.4 mg/ml for the upper layer. After polymerization of collagen gel in the bottom layer, cells were suspended in collagen gel solution. Cell suspensions were added to the dish, then immediately transferred to a 37 °C incubator for 60 min to initiate polymerization of collagen. After formation, the collagen gel was covered with culture media.

### Transendothelial migration assay

Transendothelial migration assay was performed using 24-well HTS FluoroBlok™ 8.0-μm colored PET membrane inserts (BD Biosciences). Forty-five thousand mAEC were applied to the upper chamber after coating the upper surface of the membrane with 30 μg/ml of fibronectin. After 24 h culture of mAEC, LM8 or Dunn cells were applied to the upper chamber after LM8 or Dunn cells were stained using CellTracker™ Red CMTPX according to the manufacturer’s protocol. After 12 h after application, LM8 or Dunn cells had migrated through the endothelial layer and PET membrane pores were detected using fluorescent light (excitation, 577 nm; emission, 602 nm) from the bottom side of the membrane. Attachment of LM8 or Dunn cells to mAEC and mitosis of mAEC were monitored using time-lapse photography with a Cool Snap Cf camera (Roper Scientific, Ottobrunn, Germany) and an IX70 inverted microscope (Olympus, Tokyo, Japan).

### RNA isolation, reverse transcription, and polymerase chain reaction (PCR)

Total RNA was purified using the TRIzol reagent (Invitrogen). Total RNA (1 μg) was used as a template for reverse transcription using a High-Capacity cDNA Reverse Transcription kit (Applied Biosystems, Foster City, CA) according to the instructions from the manufacturer. PCR was performed with Taq DNA Polymerase (Promega, Madison, WI) using the indicated primer sets (forward and reverse respectively) for the following genes: *Vegf*-*a*, 5′-CATGCGGATCAAACCTCAC-3′ and 5′-TTCTGGCTTTGTTCTGTCTTTC-3′; *Vegfr1*, 5′-CTCGGGTGTCTGCTTCTCA-3′ and 5′-CTCAGCCTTTTGTCCTCCTG-3′; *Vegfr2*, 5′-TTTGGCAAATACAACCCTTCAGA-3′ and 5′-GCAGAAGATACTGTCACCACC-3′ and *Gapdh*, 5′-AGGTCGGTGTGAACGGATTTG-3′ and 5′-GCAGAAGATACTGTCACCACC-3′.

### Permeability assay

The mAEC (4.5 × 10^4^) were plated in 24-well Bio-Coat cell migration chambers (diameter, 6.4 mm; pore size, 8.0 μm, BD Biosciences, Bedford, MA), grown for 24 h, and serum starved for 4 h. DMSO (2 μl), pazopanib (1 μM), VEGF (100 ng/ml) and a combination of pazopanib (1 μM) and VEGF (100 ng/ml) were added to the upper chamber (total 500 μl) containing 100 μg/ml of FITC-labeled dextran (2,000 kDa). At 0, 5, 15, 30, and 60 min, 100 μl of media was removed from the lower chamber and the amount of FITC-labeled dextran present was determined using a fluorescence spectrophotometer (1420 Multilabel Counter; PerkinElmer, Norwalk, CT). Values represent the mean of results from triplicate trials for all experiments.

### Mouse model

Specific pathogen-free 5- to 6-week-old C3H/He mice were used in this study (SLC, Shizuoka, Japan).Pazopanib solution, prepared as described previously [20], was orally administered at 100 mg/kg/day for 21 days. In order to generate a primary tumor resection model of OS, LM8 cells (1 × 10^7^ cells/200 μl of PBS) were injected into the subcutaneous tissue of the backs of syngeneic C3H mice. The primary tumor was resected under anesthesia at 11 days after injection. C3H mice were estimated as likely to die from pulmonary metastasis by 5–6 weeks after primary tumor resection. Thus, for ethical reasons, histological evaluations, count of pulmonary metastatic foci and CTCs culture were performed at 3 weeks after primary tumor resections. Serum VEGF concentration was measured using a Mouse VEGF Quantikine ELISA kit (R&D Systems), according to the instructions from the manufacturer. Serum pazopanib concentrations were measured at the institute of GlaxoSmithKline. All animal experiments were approved by the institutional animal experiments review committee, and all animals were euthanized with diethyl ether at the end of experiments.

### Phase contrast microscopy

Fluorescence and phase-contrast images were obtained with an IX70 microscope (Olympus). Images were analyzed and processed for presentation using brightness and contrast adjustment with ImageJ software (NIH Software, Bethesda, MD).

### Statistical analysis

The significance of differences was evaluated using a two-sided Student’s *t* test for biological assays, and two-sided Mann–Whitney’s *U* test for animal experiments. Values of *P* < 0.05 were considered statistically significant. All analyses were performed using Excel software (Microsoft, Redmond, WA) and Statcel3 software (OMS Publishing, Saitama, Japan).

## Results

### Chronological detection of circulating tumor DNA and living CTCs in mice with LM8 or Dunn cells, CTCs from LM8 appearance faster, rate and number greater compared to Dunn

**Fig. 1:**
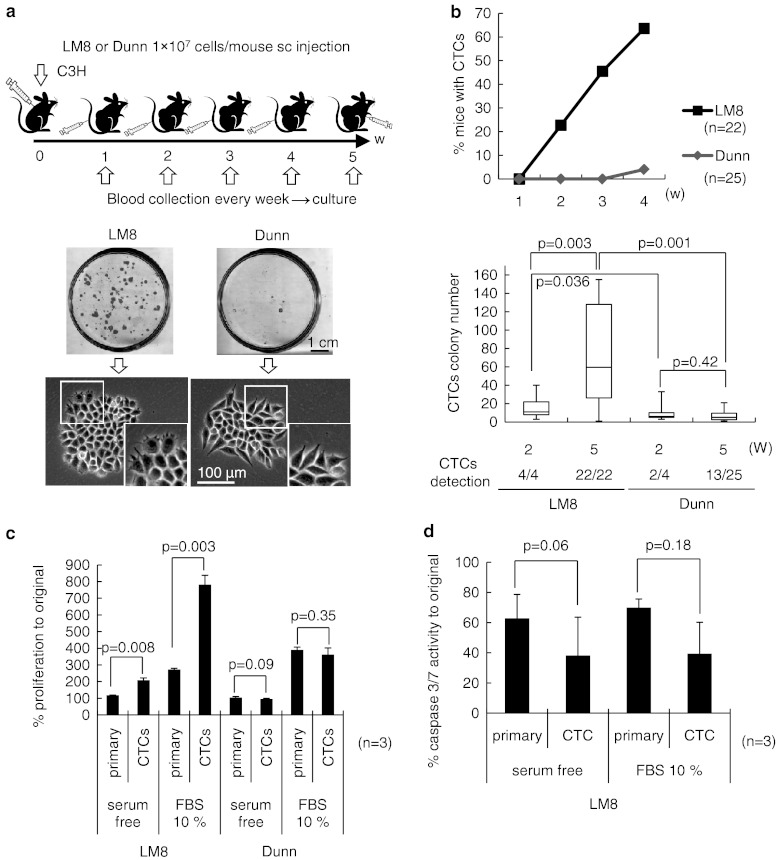
Chronological detection of living CTCs in mice with LM8 or Dunn cells, Survival ability of LM8 or Dunn cells between primary and CTCs under suspension conditions. **a** Schematic of the experimental model to detect living CTCs. On day 1, 1 × 10^7^ LM8 or Dunn cells were subcutaneous transplanted into 47 mice (LM8, *n* = 22; Dunn, n = 25). In each group, peripheral blood samples (40 μl) were collected from all mice once a week from week 1 to 4 and cultured with antibiotics. At the end of the experiment on week 5, all mice were killed and intra-cardiac blood samples were collected and cultured with antibiotics. Representative images show colony formation from living CTCs in mice with LM8 or Dunn cells. **b** The graph shows chronological detection of living CTCs in mice with LM8 or Dunn cells. Colony number of culturable CTCs per 60-mm dish from intra-cardiac blood samples were compared between week 2 and 5 in mice with both cells. Median, quartiles and highest and lowest values are indicated on box-and-whisker plots. **c** Measurement of proliferation with CellTiter Glo^®^ under suspension culture of cells from the primary site or CTCs for both cell lines. The graph shows living cell numbers at 0 and 48 h between cells from the primary site or CTCs with or without serum. **d** The graph shows caspase 3/7 activity at 0 and 48 h between LM8 from the primary site or CTCs with or without serum using Caspase-Glo^®^ under suspension culture

In order to detect circulating DNA in mice with LM8 or Dunn, we first prepared the experimental model (Supplementary Fig. 1a). Since both LM8 and Dunn cells were derived from male C3H mice, we used primers specific to the *Sry* gene on the mouse Y-chromosome as tumor DNAs and the *MyoD* gene as a control (Supplementary Fig. 1b). We performed genomic PCR of various tissues from female mice with LM8 or Dunn cells (Supplementary Fig. 1c). At week 3, almost all mice with LM8 showed positive results in lung tissue, while Dunn only showed such results at week 4. LM8 showed 25–50 % positivity in blood at week 3. Neither LM8 nor Dunn showed positivity in bone marrow. Macroscopic lung metastasis was observed after week 3 with LM8, but not with Dunn (Table 1). We detected circulating tumor DNA in mice with both cells, but could not distinguish sources as from living or dead tumor cells. Therefore, we next prepared the experimental model to detect only living CTCs. After subcutaneous injection, peripheral blood samples (40 μl) were collected from the tail vein once a week from week 1 to 5 and cultured. At the end of the experiment at week 5, all mice were killed and intra-cardiac blood samples were collected and cultured (Fig. 1a, top). Massive pulmonary metastases were seen in all mice with LM8 cells at week 5, but not in those with Dunn cells (Supplementary Fig. 2a). We were able to detect living CTCs as colonies for 2 weeks culture (Fig. 1a, bottom). To confirm whether CTC colonies showed original biological function, we checked for morphology and metastatic ability. CTCs from LM8 showed a greater number of filopodia per cell compared to those from Dunn cells (Fig. 1a, bottom), as previously reported [8]. CTCs from LM8 expanded and re-transplanted subcutaneously into another C3H mouse showed pulmonary metastasis on week 5 (Supplementary Fig. 1b), whereas CTCs from Dunn did not (data not shown). The graph showed chronological detection of living CTCs in mice with LM8 or Dunn cells (Fig. 1b, top). In LM8 cells, first detection was on week 2 in peripheral blood samples and the rate of detection gradually increased to reach 63.6 % by week 4. In Dunn cells, we could not detect CTCs until week 4 (only 4 %). We next compared the number of colonies of culturable CTCs per dish from intra-cardiac blood samples between week 2 and 5 in mice with both cells. In LM8, we could detect 100 % culturable CTCs at both times. In Dunn, detection rates were 50 % (2/4) at week 2 and 60 % (13/25) at week 5. Colony number of LM8 at week 5 was significantly higher than that at week 2 or with Dunn at week 5. At week 2, colony number was significantly higher for LM8 than for Dunn (Fig. 1b, bottom). In analyzing the chronological appearance of living CTCs, the timing of appearance was faster, and rate and number were both greater with LM8 than with Dunn.

**Table 1:** Genomic PCR between LM8 highly metastatic cells and parental Dunn cells genomic PCR from various tissues in female mice with LM8 or Dunn cells

	Week	Genomic PCR				Macroscopic lung metastasis
		Primary tumor	Lung	Blood	BM	
LM8	2	3/3	1/3	0/3	0/3	0/3
	3	4/4	4/4	1/4	0/4	1/4
	4	4/4	3/4	1/4	0/4	3/4
	5	2/2	2/2	1/2	0/2	2/2
Dunn	2	3/3	0/3	0/3	0/3	0/3
	3	3/3	0/3	0/3	0/3	0/3
	4	3/3	1/3	0/3	0/3	0/3
	5	3/3	0/3	0/3	0/3	0/3

Primary tumors of all mice with LM8 or Dunn cells show positivity using primers for the Y-chromosome specific gene, *Sry*. At 3 weeks after injection, almost all mice with LM8 show positive results in lung tissue, but mice with Dunn showed positive results in 2/3 at 4 weeks and 1/3 at 5 weeks. Only mice with LM8 show 25–50 % positivity in blood at 3 weeks after injection. Neither LM8 nor Dunn show positivity in bone marrow. Macroscopic lung metastasis was detectable at 3 weeks after injection with LM8, but not with Dunn. *BM* bone marrow

### Culture of LM8 and Dunn cells in suspension, CTCs from LM8 showed greater proliferation compared to primary site

In order to check the next step of metastasis, survival in the bloodstream, we performed a suspension culture mimicking the situation of CTCs in blood vessels. We prepared a HEMA-coated dish with non-adherent conditions to mimic the floating cell state. After 2 days, both cell lines showed only floating-state cells on non-adherent dishes (Supplementary Fig. 3, lower left), and only attached-state cells on Petri dishes (Supplementary Fig. 3, lower right). We then analyzed the proliferation and anti-anoikis ability between primary site and CTCs in suspension condition. In LM8 cells, proliferation of CTCs was significantly higher than that of primary site in both serum-free and 10 % FBS media (Fig. 1c). By contrast, Dunn cells showed no difference in proliferation between primary and CTCs (Fig. 1c). In LM8 cells, a comparison of caspase 3/7 activity in both primary and CTCs showed no difference in activity (Fig. 1d). These data indicated that CTCs from mice with LM8 acquired greater proliferation ability through intravasation step and survival in circulation.

### Three dimensional culture of LM8 or Dunn cells under different stiffness conditions, LM8 preferred to lung-mimicking condition

**Fig. 2:**
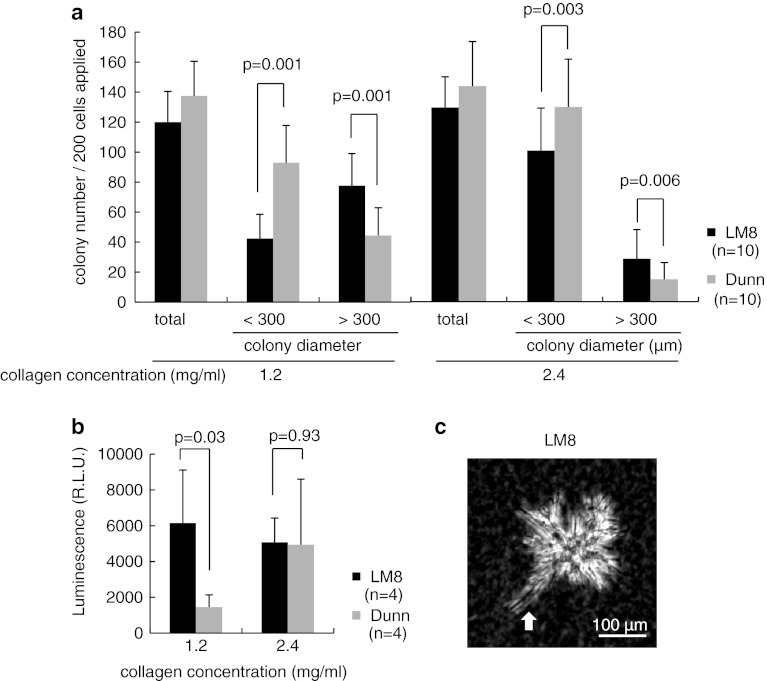
Three Dimensional culture of LM8 or Dunn cells under conditions of differing stiffness. **a** Two-hundred LM8 or Dunn cells were cultured with collagen 1 gel of different concentrations (1.2 or 2.4 mg/ml) in 35-mm dishes for 7 days. The graph shows total colony number, and colony diameter <300 μm and >300 μm of LM8 or Dunn cells under different stiffness conditions. **b** Forty LM8 or Dunn cells were cultured with collagen 1 gel of different concentrations (1.2–2.4 mg/ml) in 96 wells for 7 days. Proliferations of both cells were measured with CellTiter-Glo^®^. **c** LM8 cells were cultured for 5 days with 1.2 mg/ml collagen 1 gel concentration. Supplemental movie 1 shows LM8 cells detached from the original colony

We next checked colony formation step in distant organs. We prepared three-dimensional cultures with various stiffness conditions using different concentrations of collagen 1 gel (1.2 and 2.4 mg/ml). The 1.2 mg/ml collagen 1 gel stiffness mimics the lung environment (approximately 150 Pa), while 2.4 mg/ml collagen 1 gel stiffness mimics the soft tissue environment (~800–1,200 Pa) [21, 22]. Two-hundred LM8 or Dunn cells were cultured with collagen 1 gel at different concentrations (1.2 and 2.4 mg/ml) for 7 days. Total colony number, and colony diameters <300 μm and >300 μm of LM8 or Dunn cells under different stiffness condition were determined. No differences in total colony number were seen between LM8 and Dunn cells under different stiffness conditions. However, LM8 cells showed bigger colonies than Dunn cells under stiffness conditions mimicking the lung environment (Fig. 2a). We then checked whether there were differences in the number of living cells or only differences in morphology. We were able to measure the number of living cells in collagen 1 gel with CellTiter-Glo^®^ (Supplementary Fig. 4). No differences in living cell number in colonies were seen between LM8 and Dunn cells with 7-day culture in 2.4 mg/ml collagen 1 gel, but proliferation of LM8 was higher than that of Dunn cells in 1.2 mg/ml collagen 1 gel (Fig. 2b). LM8 cells were cultured for 5 days with 1.2 mg/ml collagen 1 gel concentration (Fig. 2c). A supplemental movie at 10 h shows LM8 cells detached from the original colony (Supplemental movie 1), but Dunn cells did not (data not shown). These data indicated proliferation of LM8 cells was faster and colony size was bigger under lung-mimicking conditions than under stiffer conditions.

### Transendothelial migration of LM8 showed higher than that of Dunn cells

**Fig. 3:**
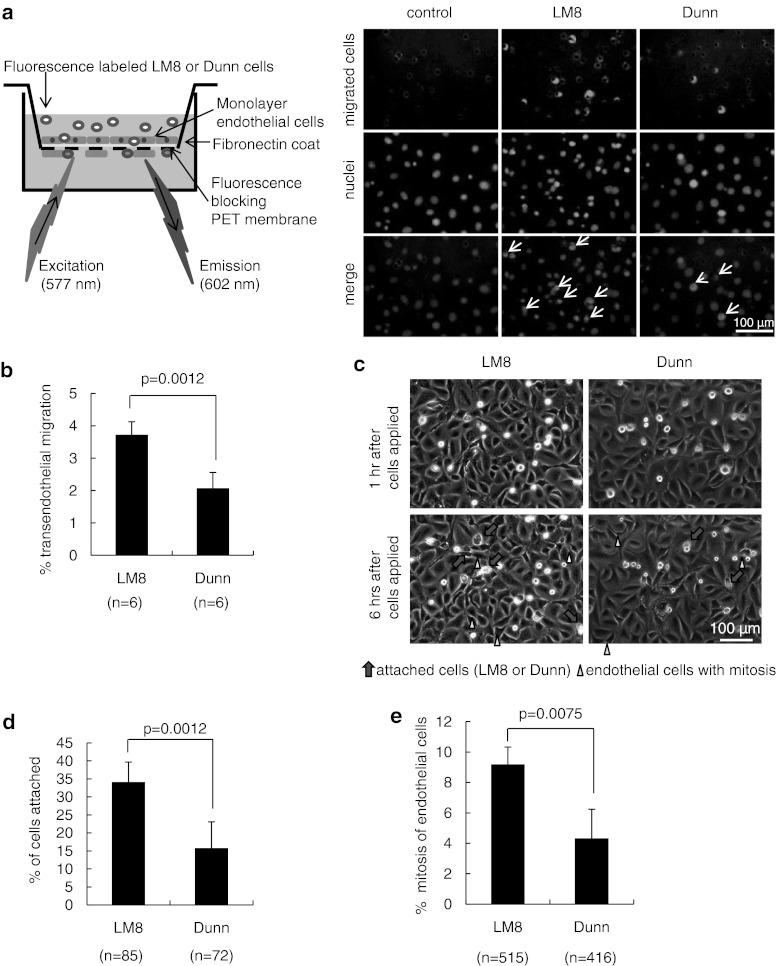
Transendothelial migration of LM8 and Dunn cells. **a** Schematic presentation of the transendothelial migration assay. Fluorescence-labeled LM8 or Dunn cells applied onto the upper chamber with a monolayer of cultured mAEC on a fibronectin-coated fluorescence-blocking PET membrane. After 12 h, LM8 or Dunn cells that had migrated through the endothelial layer and PET membrane pores were detected using fluorescent light from the bottom side of the membrane (*right panel, top row*). Nuclei of migrated LM8, Dunn and endothelial cells are shown (*middle row*). In merged images (*bottom row*, *white arrows*) indicate migrated LM8 or Dunn cells. **b** The percentage of transendothelial migration of LM8 and Dunn. **c** Fluorescence-labeled LM8 or Dunn cells applied onto monolayer mAEC. Morphological changes in tumor and endothelial cells were recorded 1–6 h after application using time-lapse imaging. **d** The percentage of tumor cells attached to endothelial cells for LM8 and Dunn cells up to 5 h. **e** The percentage of endothelial cell mitoses in LM8 and Dunn cells up to 5 h

Since LM8 showed higher migration ability compared to Dunn, we next checked the extravasation step with a transendothelial migration assay (Fig. 3a, left). The mAEC were prepared as a monolayer on fibronectin-coated fluorescence-blocking PET membrane (Supplementary Fig. 5a, left). We then macroscopically examined monolayer endothelial cells (Supplementary Fig. 5a, right) and checked biological barrier function with a permeability assay (Supplementary Fig. 5b). At 12 h after seeding, LM8 or Dunn cells had migrated through the endothelial layer and PET membrane pores were detected using fluorescent light (Fig. 3a, right). No different migration ability was seen between original and fluorescence-labeled LM8 or Dunn cells (Supplementary Fig. 5c). The transendothelial migration ability of LM8 was significantly higher than that of Dunn cells (Fig. 3b). To elucidate how tumor cells migrated into endothelium, we recorded them with time-lapse imaging. Morphological changes of tumor and mAEC were analyzed between 1 and 6 h after application (Fig. 3c; Supplementary movies 2, 3). The percentage of tumor cells attached to mAEC was significantly higher for LM8 than for Dunn cells up to 5 h (Fig. 3d). The percentage of mitoses in mAEC was also significantly higher with LM8 than with Dunn cells during these 5 h (Fig. 3e). These data indicate that LM8 not only showed significantly higher migration ability, but also induced significantly higher rates of mitosis in mAEC than Dunn cells.

### LM8 secrete higher levels of VEGF than Dunn, and pazopanib inhibits VEGF–VEGFR signals for extravasation step

**Fig. 4:**
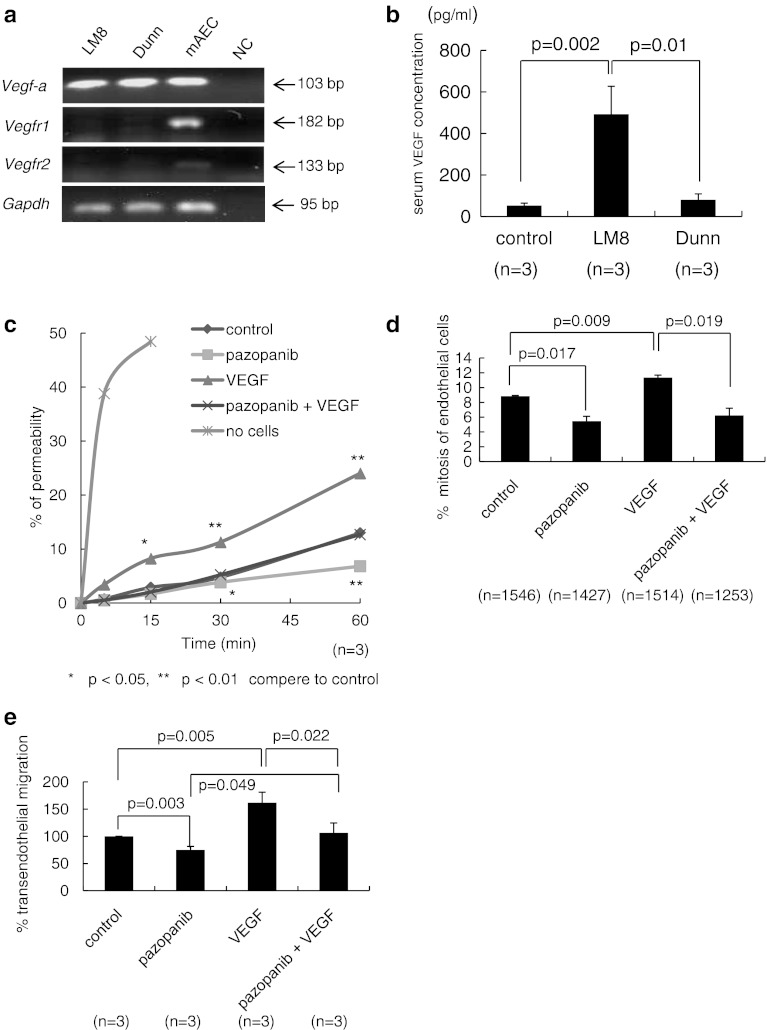
LM8 secrete VEGF more than Dunn, and pazopanib inhibits VEGF signals. **a** RT-PCR analysis of the mRNA expression of VEGF-A, VEGF receptor 1 (VEGFR1), VEGF receptor 2 (VEGFR2) and GAPDH as a control. **b** Serum VEGF concentrations were measured in mice with LM8, Dunn and no cells using ELISA. **c** After culture for 24 h of 45,000 endothelial cells, FITC-Dextran (molecular weight, 2,000 kDa; 100 μg/ml) treated with control, pazopanib 1 μM, VEGF 100 ng/ml of the combination pazopanib of 1 μM and VEGF 100 ng/ml added into the upper well, FITC intensity of the lower chamber medium was measured at 5, 15, 30, and 60 min. **d** The percentage of mitoses among endothelial cells treated with control, pazopanib 1 μM, VEGF 100 ng/ml or the combination of pazopanib 1 μM and VEGF 100 ng/ml during 5 h. **e** Percentages of transendothelial migration for LM8 treated with control, pazopanib 1 μM, VEGF 100 ng/ml and the combination of pazopanib 1 μM and VEGF 100 ng/ml

We analyzed mRNA expression of VEGF-A and its receptors VEGFR1 and VEGFR2 in LM8, Dunn and mAEC. VEGF-A was shown in all cell lines, but VEGFR1 and VEGFR2 were only observed in mAEC (Fig. 4a). Therefore, VEGF secreted from tumor cells could only interact with VEGFRs of mAEC. Mean serum VEGF concentrations were 52.9 ± 12.0 pg/ml for control mice, 493.2 ± 133.7 pg/ml for LM8 and 80.8 ± 28.5 pg/ml for Dunn-bearing mice (Fig. 4b). Since VEGF has been reported as vascular permeability factor, we first checked the vascular permeability effect of VEGF and its inhibitor VEGFR-TKI (pazopanib). The mAEC grown on Boyden chamber tissue culture inserts form a tight cell monolayer that blocks the passage of macromolecules and has functional barrier. The functional barrier of mAEC was reinforced by pazopanib within 30 min, and VEGF-induced mAEC permeability to high molecular weight dextran (2,000 kDa) was prevented by pazopanib within 15 min (Fig. 4c). Next, to elucidate the mechanism of VEGF-induced mAEC paracellular permeability, we analyzed mAEC behavior using time-lapse imaging, and then noticed extending cell–cell gap during mAEC mitosis. The percentage of mitoses among mAEC treated with pazopanib was significantly lower than that of treated with control, and we noticed VEGF-induced percentage of mitoses was significantly decreased by pazopanib. Thus, extending cell–cell gaps between mAECs induced by VEGF made increasing endothelium permeability, and pazopanib inhibited the effect by decreasing the percentage of mitoses. Last, as shown Fig. 4e, the transendothelial migration ability of LM8 treated with pazopanib was significantly lower than that with control, and VEGF-induced transendothelial migration ability blocked by pazopanib. Since no different migration ability using Boyden chamber was seen between LM8 treated with control, pazopanib, VEGF, and the combination of pazopanib and VEGF (Supplementary Fig. 6), these data indicate that VEGF-induced transendothelial migration by accelerating mAEC permeability which extending cell–cell gaps made tumor cells passing endothelium easily, and pazopanib inhibited the effect.

### Pazopanib inhibits pulmonary metastasis in syngeneic mouse models

**Fig. 5:**
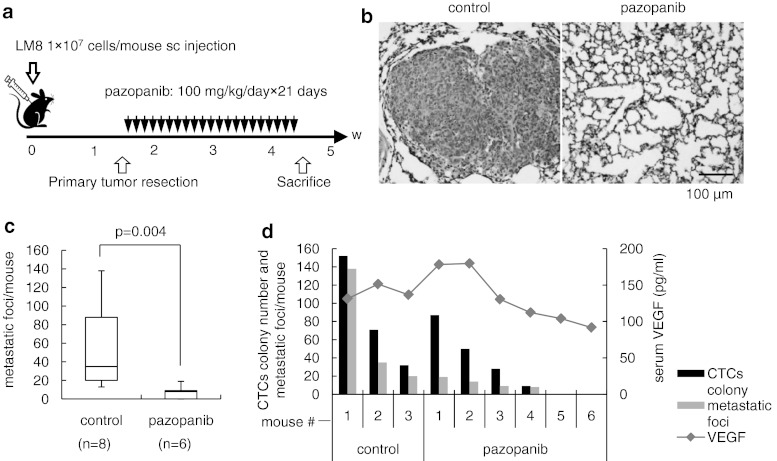
Continuous oral administration of pazopanib inhibited pulmonary metastasis in syngeneic mouse models. **a** For the primary tumor resection model, a total of 14 mice were divided into 2 groups: (i) untreated controls (8 mice); (ii) treated with pazopanib at 100 mg/kg/day for 21 days (6 mice). On day 1, 1 × 10^7^ LM8 cells were transplanted subcutaneously into each mouse. Primary tumor nodules were surgically removed on day 11 and oral administration of pazopanib was started from day 12. All mice were killed on day 33. **b** Histological images of pulmonary metastasis are shown (HE staining). In the control group, LM8 cells formed large nodules and invaded into lung alveoli (*left*). In the treatment group, alveoli architecture was preserved and tumor nodules were not shown (*right*). **c** Number of metastatic foci in lungs per mouse. Median, quartiles and highest and lowest values are indicated on box-and-whisker plots. **d** This graph showed CTC colony number per 40 μl of peripheral blood, metastatic foci per mouse and serum VEGF concentration

We finally checked whether pazopanib show the effectiveness or not in vivo. We prepared a primary tumor resection model, in which pulmonary metastasis was not obvious at the time of primary tumor resection but became apparent at 3 weeks after resection. This model fits rather clinical status because of mimicking after operation. No significant difference in tumor size was apparent between treatment and control groups at tumor resection. As shown in Fig. 5a, oral administration of pazopanib started after primary tumor resection (100 mg/kg/day × 21 days). No loss of body weight was seen with the administration of pazopanib. Pazopanib administration was associated with a substantial decrease in pulmonary metastasis. In the control group, histological images showed large metastatic foci that infiltrated into lung parenchyma, whereas metastatic foci were much smaller and solitary in the treatment group (Fig. 5b). The number of metastatic foci in the lungs per mouse was significantly lower in the treatment group (Fig. 5c). At the end of the experiment on day 33, all mice were killed and intra-cardiac blood samples were collected and cultured with antibiotics. The percentage of mice in which CTCs were detectable was 100 % (8/8) among controls and 66.7 % (4/6) in the 100 mg/kg/day group. Figure 5d showed the correlation between CTCs colony number, metastatic foci, and serum VEGF concentration. In pazopanib treatment group, parallel correlation was observed between these three factors.

## Discussion

In this study, we reported that LM8 displayed several superior biological characteristics in all metastatic steps rather than just a single step, by assessing the details of the metastatic cascade in a step-by-step manner using experimental systems to mimic in vivo conditions.

Previous studies have reported that isolation or detection of living CTCs was too difficult, because phenotype would be changed between primary site and CTCs in their quite different microenvironments, so the characteristics of these cells have remained unclear [7, 12, 13]. In this study, we were able to culture living CTCs chronologically not only from mice with LM8 but also with Dunn. These results corresponded to the clinical reports that showed identification of CTCs in the peripheral blood of patients with early-stage localized prostate and breast cancer, as well as metastatic cancers using CellSearch systems (Veridex, North Raritan, NJ), as correlating with response to chemotherapy in patients with metastatic cancer [23–29]. These methods measured only CTCs number but not analyzed its characterization. By contrast, our method could detect not only living CTCs but also analyze biological feature—higher proliferation ability of CTCs from LM8 compared to cells from primary site in suspension culture, while CTCs from Dunn did not show such a character. So far, there is a fear that phenotype of CTCs may be changed from original status while culturing in 2D condition. To clarify the precise mechanism, further improvement and combination with single cell analysis method would be required.

During the colonization step, LM8 also showed higher proliferation ability compared to Dunn in conditions that mimicked in lung environment (−150 Pa), but not in stiffer condition (800–1,200 Pa) [21, 22]. In a mouse fibrosarcoma model, the proliferation rate of highly metastatic potential cell lines was higher than that of non-metastatic or intermediate potential cell lines in stiffer condition [30]. Thus, our result opposed the previous report. The precise mechanism which LM8 preferred lung-mimicking condition than Dunn was unknown, and further examination should be required to clarify the mechanism.

The physiological role of VEGF has been reported that neovascularization during embryonic development, skeletal growth, and reproduction. In addition, overexpression of VEGF in tumor could stimulate vasculogenic and angiogenic switches [14]. We have previously shown that VEGF level secreted from LM8 was higher than that from Dunn [8]. Here, we speculated that VEGF from tumor cells increased number of endothelial cell mitosis, resulting in opening cell–cell junction among endothelial cells, following to enhanced transendothelial migration. In fact, transendothelial migration was reduced by blocking the VEGF–VEGFR signal with pazopanib (Votrient; GlaxoSmithKline), multiple TKI targeting VEGFRs [20]. This compound has been approved by the FDA for the treatment of advanced renal cell carcinoma (2009) and soft tissue sarcoma (2011). We first checked the effect of pazopanib in vivo tumor bearing mouse model (Supplementary Fig. 7). Administration of pazopanib significantly reduced only metastatic foci in lung without affecting primary site growth. To elucidate the effect on lung metastasis in clinical status more clearly, we next prepared a primary tumor resection model (Fig. 5a). We finally confirmed that pazopanib administration significantly reduced lung metastatic foci and the parallel correlation among CTCs colony number, metastatic foci and serum VEGF concentration. In this experiment, mean serum pazopanib concentrations were 106.1 ± 37.4 μM at 8–9 h after final oral administration. In terms of human pharmacokinetics, daily dosing at 800 mg results in a geometric mean C_max_ of 132 μM. Thus, our experimental dose of pazopanib was comparable to the dose used clinically in humans.

Lastly, we focused on parallel correlations among lung metastases, the number of CTCs and serum VEGF concentration. The source of VEGF was considered based on the following three points in this experiment. First, at the time of primary tumor resection, median colony number 11 per 40 μl blood sample (Fig. 1b, bottom), thus the number of CTCs in whole blood (~1 ml) was estimated ~275. It is unlikely that VEGF secreted from only 275 cells influence total concentration of VEGF in blood. Second, because pazopanib did not show anti-tumor effects under suspension conditions (Supplementary Fig. 8), CTCs might be died in circulation before CTCs overcome the extravasation and colonization steps. In fact, Pantel and Brakenhoff [31] have reported that CTCs survived for only a short period of time. Third, the existence of tumor burden is important for CTCs as tumor cell resource. Several reports have been shown that the bone marrow as an important reservoir of tumor cells for many types of malignancy [31, 32]. However, we did not detect tumor cells in bone marrow in mice with LM8 even in 5 weeks after tumor transplantation (Table 1). Thus, metastatic sites would be considered as a tumor cell resource, and might supply new CTCs and VEGF. Based on these inferences, (1) CTCs could not pass through endothelium (extravasation) by blocking VEGF–VEGFR interaction using pazopanib, (2) reducing metastatic foci in lung, (3) decreasing amount of VEGF and number of CTCs from metastatic site, (4) blocking extravasation step as vicious cycle.

So far, VEGF–VEGFR blockage therapies using monoclonal antibodies or small molecules approved by the FDA for the treatment of various cancers have proven effective for extending progression-free survival [33–35]. On the other hand, increasing micro-metastasis in certain cancers has been arisen as an issue of great concern [36, 37], because previous preclinical drug development did not consider the impact on metastasis [38]. In this study, we propose that anti-VEGF therapy using small molecules could offer a potentially useful strategy for inhibiting transendothelial migration in vitro and lung metastasis in vivo from OS.

## Electronic supplementary material

Below is the link to the electronic supplementary material.

Supplementary material 1 (PPTX 172 kb)

Supplementary material 2 (PPTX 453 kb)

Supplementary material 3 (PPTX 443 kb)

Supplementary material 4 (PPTX 43 kb)

Supplementary material 5 (PPTX 184 kb)

Supplementary material 6 (PPTX 35 kb)

Supplementary material 7 (PPTX 58 kb)

Supplementary material 8 (PPTX 36 kb)

Supplementary material 9 (MP4 2087 kb)

Supplementary material 10 (MP4 1292 kb)

Supplementary material 11 (MP4 1277 kb)

## Abbreviations

CTCs: Circulating tumor cells
3D: Three dimensions
VEGF: Vascular endothelial growth factor
