# Supplementary material for: Dynamic analysis of lung metastasis by mouse osteosarcoma LM8: VEGF is a candidate for anti-metastasis therapy
Source: Clin Exp Metastasis. 2012 Oct 18;30(4):369–79. doi: 10.1007/s10585-012-9543-8 (PMC3616224; doi:10.1007/s10585-012-9543-8)
Supplement: Supplementary file 1 — Supplementary material 1 (PPTX 172 kb) [file 10585_2012_9543_MOESM1_ESM.pptx]

## Slide 1
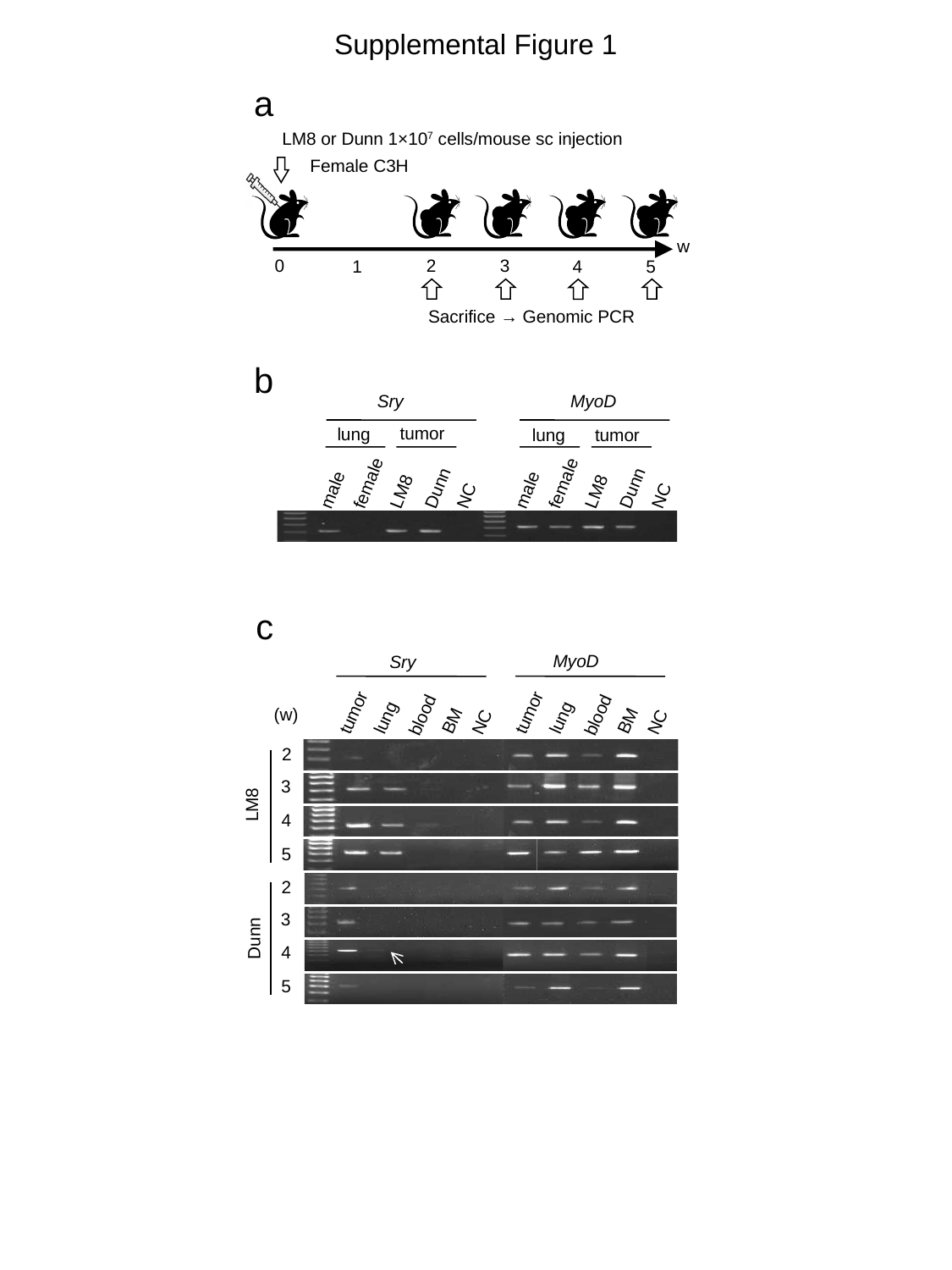

Supplemental Figure 1
a
LM8 or Dunn 1×10^7^ cells/mouse sc injection
Female C3H
w
0
2
3
1
4
5
Sacrifice → Genomic PCR
b
MyoD
Sry
tumor
lung
tumor
lung
male
LM8
Dunn
NC
male
LM8
Dunn
NC
female
female
c
MyoD
Sry
tumor
tumor
NC
NC
blood
blood
BM
lung
lung
BM
(w)
2
3
LM8
4
5
2
3
4
5
Dunn
