# Supplementary material for: Dynamic analysis of lung metastasis by mouse osteosarcoma LM8: VEGF is a candidate for anti-metastasis therapy
Source: Clin Exp Metastasis. 2012 Oct 18;30(4):369–79. doi: 10.1007/s10585-012-9543-8 (PMC3616224; doi:10.1007/s10585-012-9543-8)
Supplement: Supplementary file 2 — Supplementary material 2 (PPTX 453 kb) [file 10585_2012_9543_MOESM2_ESM.pptx]

## Slide 1
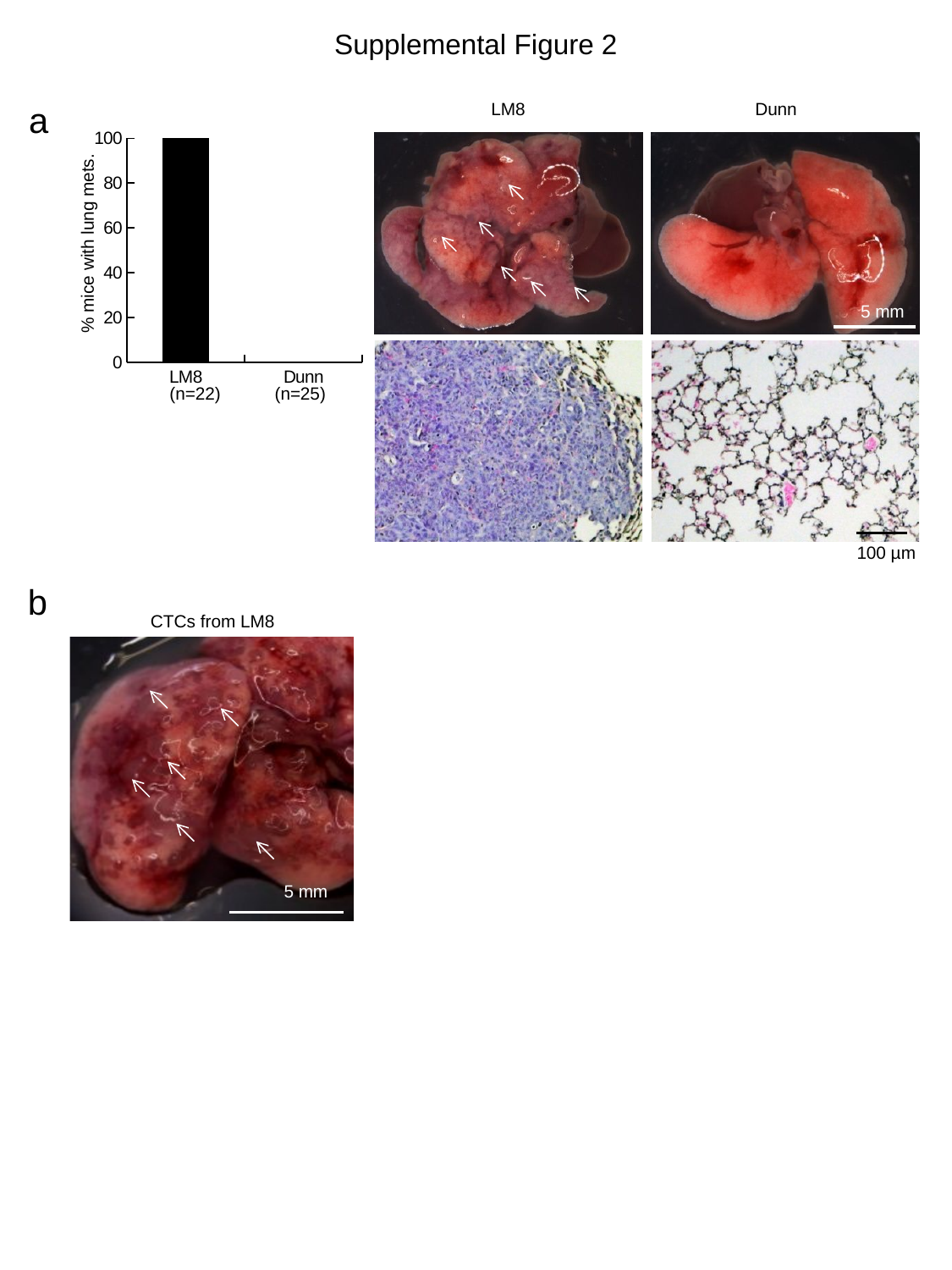

Supplemental Figure 2
Dunn
LM8
5 mm
100 µm
a
### Chart
| Category | |
|---|---|
| LM8 | 100.0 |
| Dunn | 0.0 |% mice with lung mets.
(n=22)
(n=25)
b
CTCs from LM8
5 mm
