# Supplementary material for: Dynamic analysis of lung metastasis by mouse osteosarcoma LM8: VEGF is a candidate for anti-metastasis therapy
Source: Clin Exp Metastasis. 2012 Oct 18;30(4):369–79. doi: 10.1007/s10585-012-9543-8 (PMC3616224; doi:10.1007/s10585-012-9543-8)
Supplement: Supplementary file 3 — Supplementary material 3 (PPTX 443 kb) [file 10585_2012_9543_MOESM3_ESM.pptx]

## Slide 1
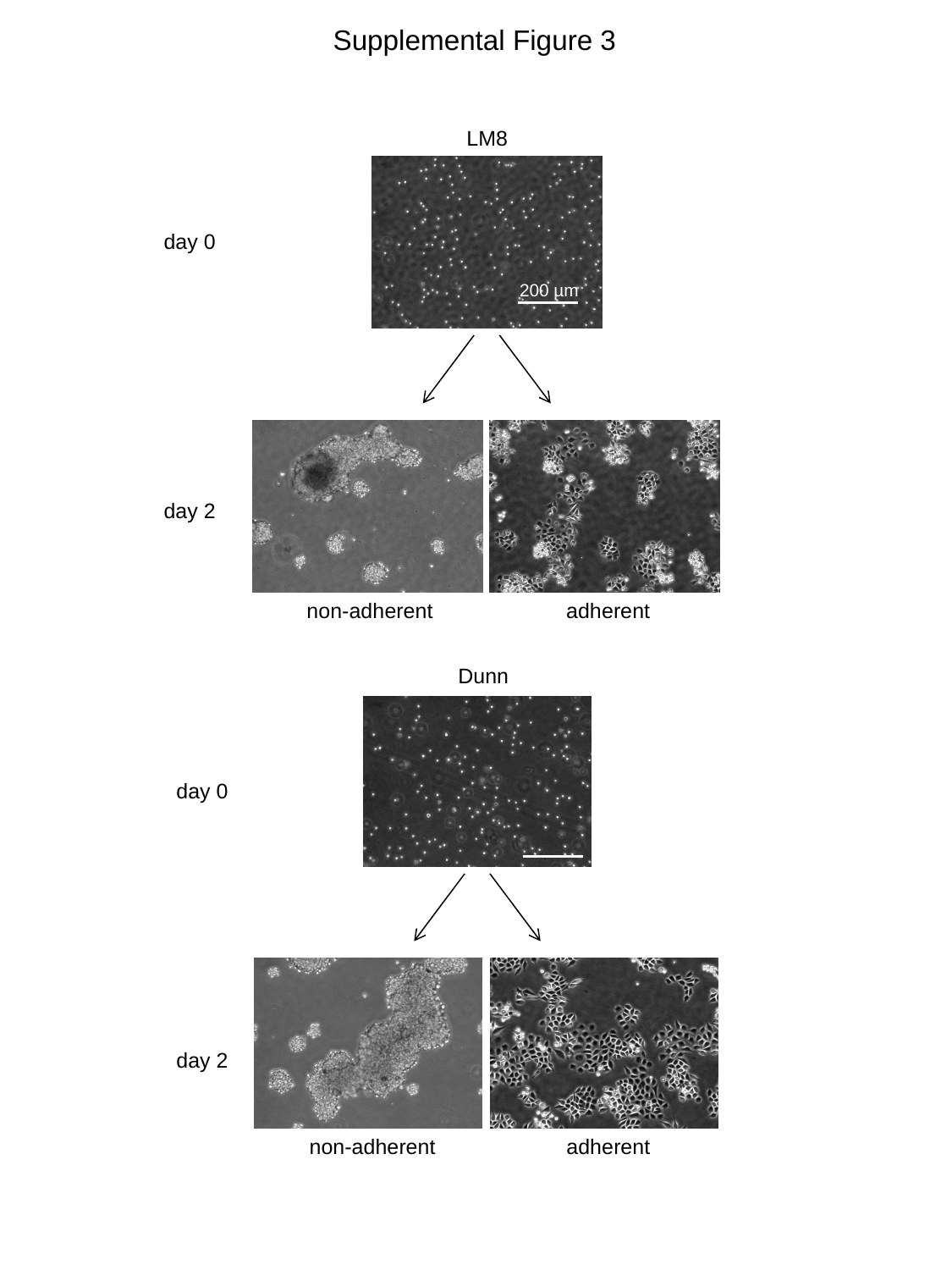

Supplemental Figure 3
LM8
non-adherent
adherent
day 0
200 µm
day 2
Dunn
non-adherent
adherent
day 0
day 2
