# Supplementary material for: Dynamic analysis of lung metastasis by mouse osteosarcoma LM8: VEGF is a candidate for anti-metastasis therapy
Source: Clin Exp Metastasis. 2012 Oct 18;30(4):369–79. doi: 10.1007/s10585-012-9543-8 (PMC3616224; doi:10.1007/s10585-012-9543-8)
Supplement: Supplementary file 4 — Supplementary material 4 (PPTX 43 kb) [file 10585_2012_9543_MOESM4_ESM.pptx]

## Slide 1
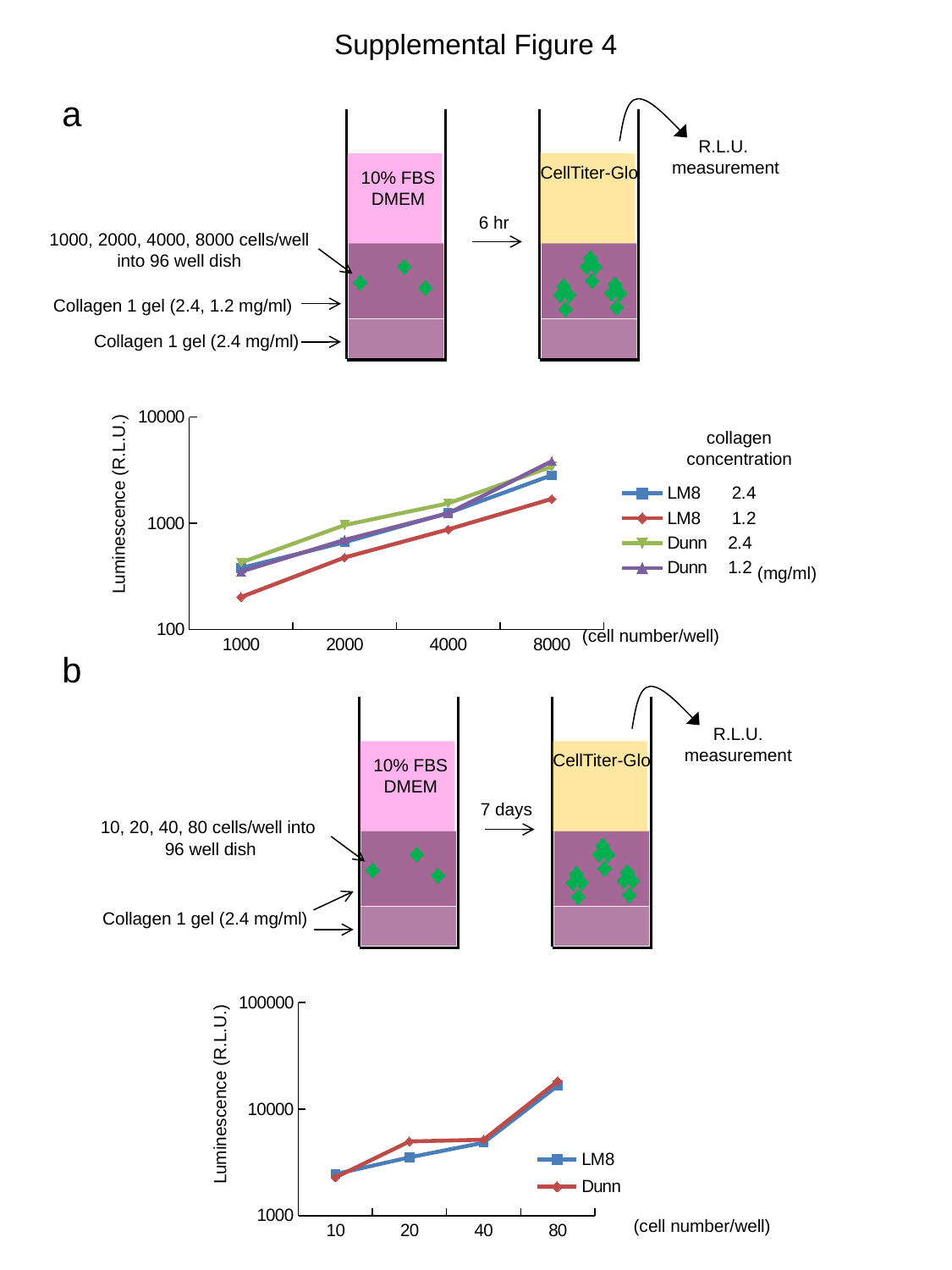

Supplemental Figure 4
a
10% FBS DMEM
Collagen 1 gel (2.4, 1.2 mg/ml)
Collagen 1 gel (2.4 mg/ml)
CellTiter-Glo
R.L.U. measurement
6 hr
1000, 2000, 4000, 8000 cells/well into 96 well dish
### Chart
| Category | LM8 2.4 | LM8 1.2 | Dunn 2.4 | Dunn 1.2 |
|---|---|---|---|---|
| 1000 | 379.0 | 202.0 | 427.0 | 351.0 |
| 2000 | 663.0 | 476.0 | 962.0 | 697.0 |
| 4000 | 1250.0 | 876.0 | 1542.0 | 1240.0 |
| 8000 | 2834.0 | 1697.0 | 3413.0 | 3838.0 |Luminescence (R.L.U.)
collagen
concentration
(mg/ml)
(cell number/well)
b
10% FBS DMEM
Collagen 1 gel (2.4 mg/ml)
CellTiter-Glo
R.L.U. measurement
7 days
10, 20, 40, 80 cells/well into
96 well dish
### Chart
| Category | LM8 | Dunn |
|---|---|---|
| 10 | 2454.4 | 2280.25 |
| 20 | 3517.6923076923076 | 4962.769230769342 |
| 40 | 4831.25 | 5154.5 |
| 80 | 16592.28571428572 | 18281.428571428572 |(cell number/well)
Luminescence (R.L.U.)
