# Supplementary material for: Dynamic analysis of lung metastasis by mouse osteosarcoma LM8: VEGF is a candidate for anti-metastasis therapy
Source: Clin Exp Metastasis. 2012 Oct 18;30(4):369–79. doi: 10.1007/s10585-012-9543-8 (PMC3616224; doi:10.1007/s10585-012-9543-8)
Supplement: Supplementary file 5 — Supplementary material 5 (PPTX 184 kb) [file 10585_2012_9543_MOESM5_ESM.pptx]

## Slide 1
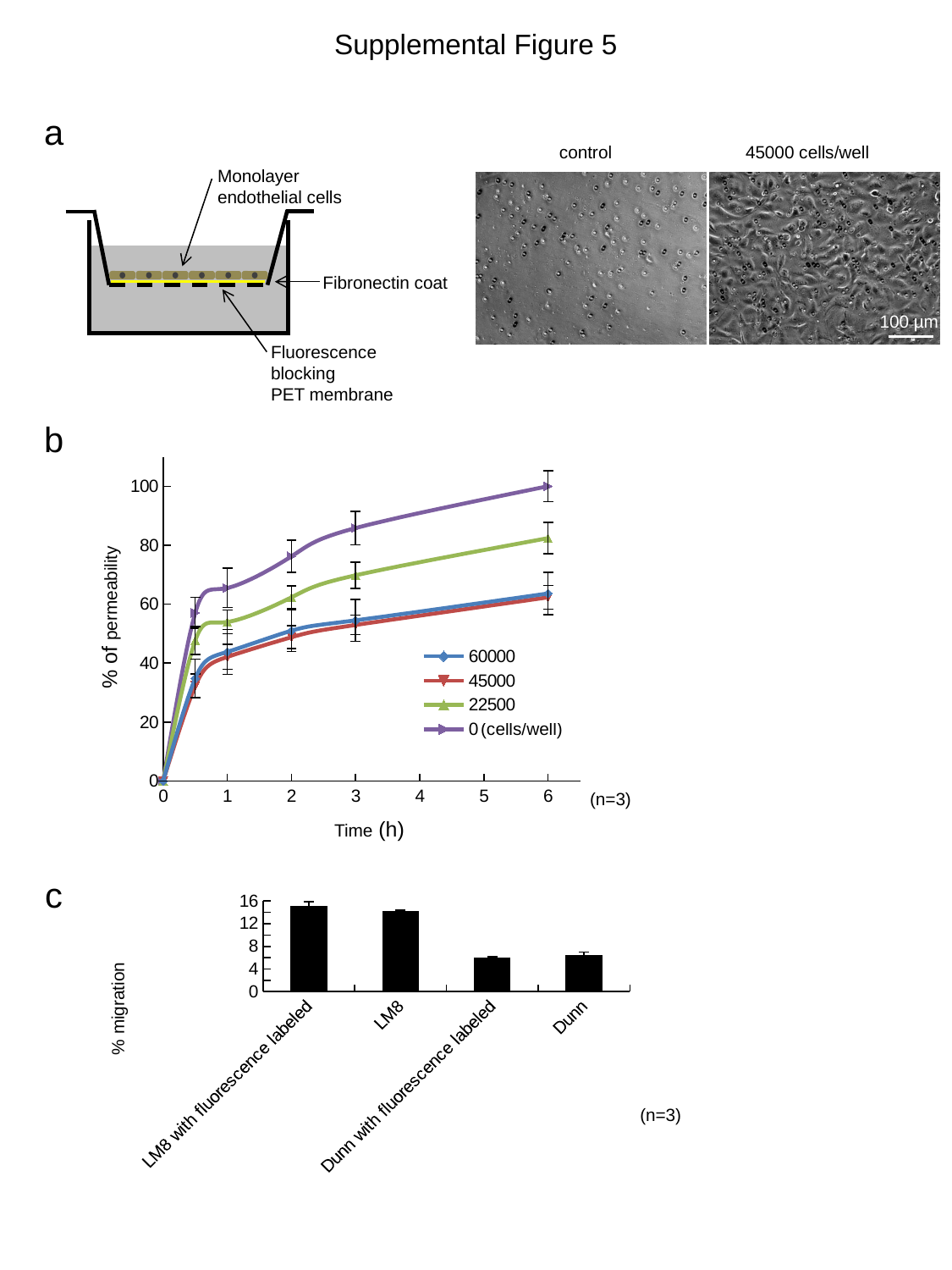

Supplemental Figure 5
a
control
45000 cells/well
100 µm
Monolayer
endothelial cells
Fibronectin coat
Fluorescence
blocking
PET membrane
b
### Chart
| Category | 60000 | 45000 | 22500 | 0 |
|---|---|---|---|---| % of permeability
Time (h)
(cells/well)
(n=3)
c
### Chart
| Category | % migrated cell |
|---|---|
| LM8 with fluorescence labeled | 15.096 |
| LM8 | 14.256 |
| Dunn with fluorescence labeled | 6.048 |
| Dunn | 6.504 |% migration
(n=3)
