# Supplementary material for: Dynamic analysis of lung metastasis by mouse osteosarcoma LM8: VEGF is a candidate for anti-metastasis therapy
Source: Clin Exp Metastasis. 2012 Oct 18;30(4):369–79. doi: 10.1007/s10585-012-9543-8 (PMC3616224; doi:10.1007/s10585-012-9543-8)
Supplement: Supplementary file 6 — Supplementary material 6 (PPTX 35 kb) [file 10585_2012_9543_MOESM6_ESM.pptx]

## Slide 1
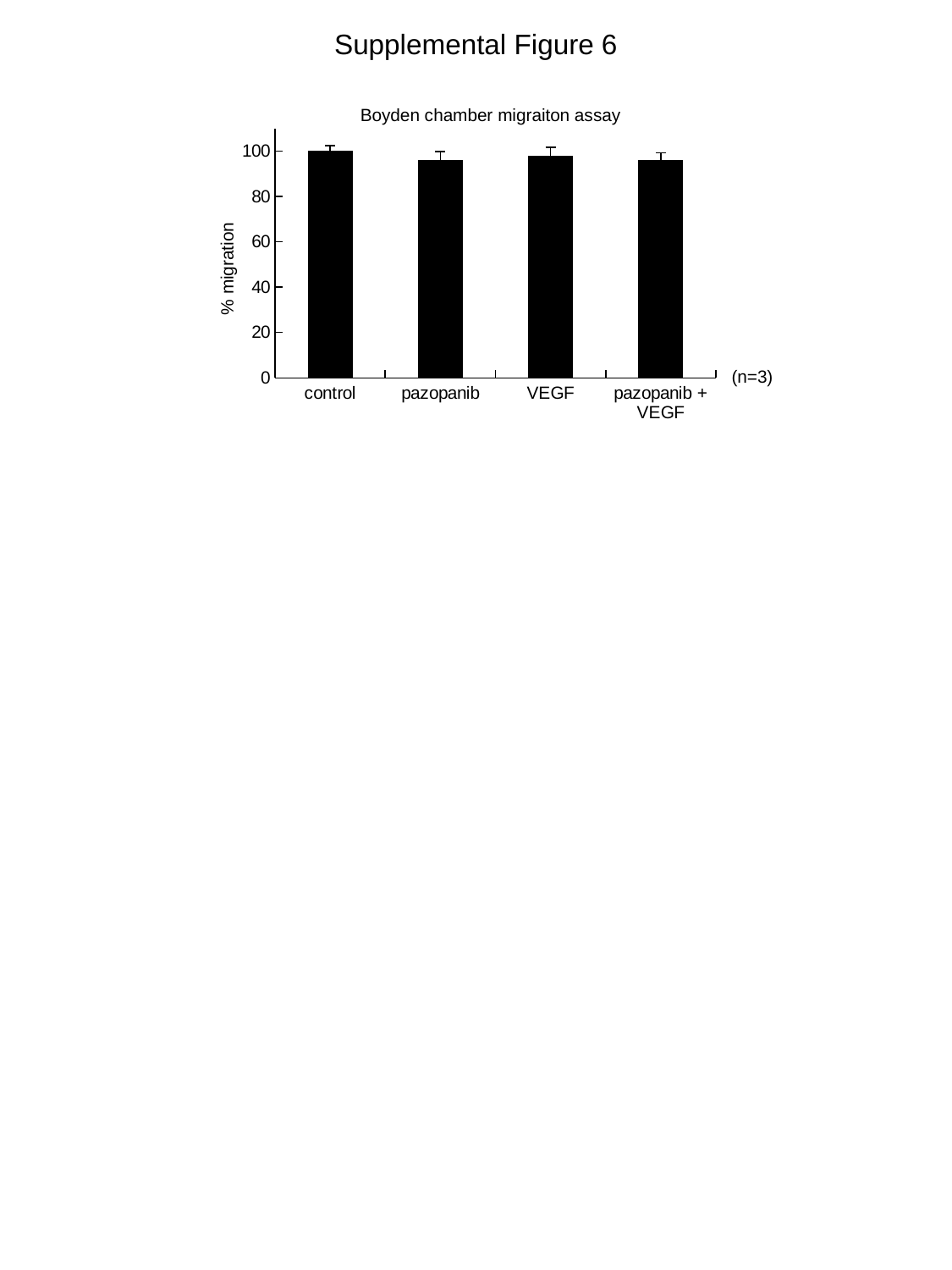

Supplemental Figure 6
Boyden chamber migraiton assay
### Chart
| Category | |
|---|---|
| control | 100.0 |
| pazopanib | 95.80312019336411 |
| VEGF | 97.7807075368045 |
| pazopanib + VEGF | 96.02285212041257 |% migration
(n=3)
