# Supplementary material for: Dynamic analysis of lung metastasis by mouse osteosarcoma LM8: VEGF is a candidate for anti-metastasis therapy
Source: Clin Exp Metastasis. 2012 Oct 18;30(4):369–79. doi: 10.1007/s10585-012-9543-8 (PMC3616224; doi:10.1007/s10585-012-9543-8)
Supplement: Supplementary file 7 — Supplementary material 7 (PPTX 58 kb) [file 10585_2012_9543_MOESM7_ESM.pptx]

## Slide 1
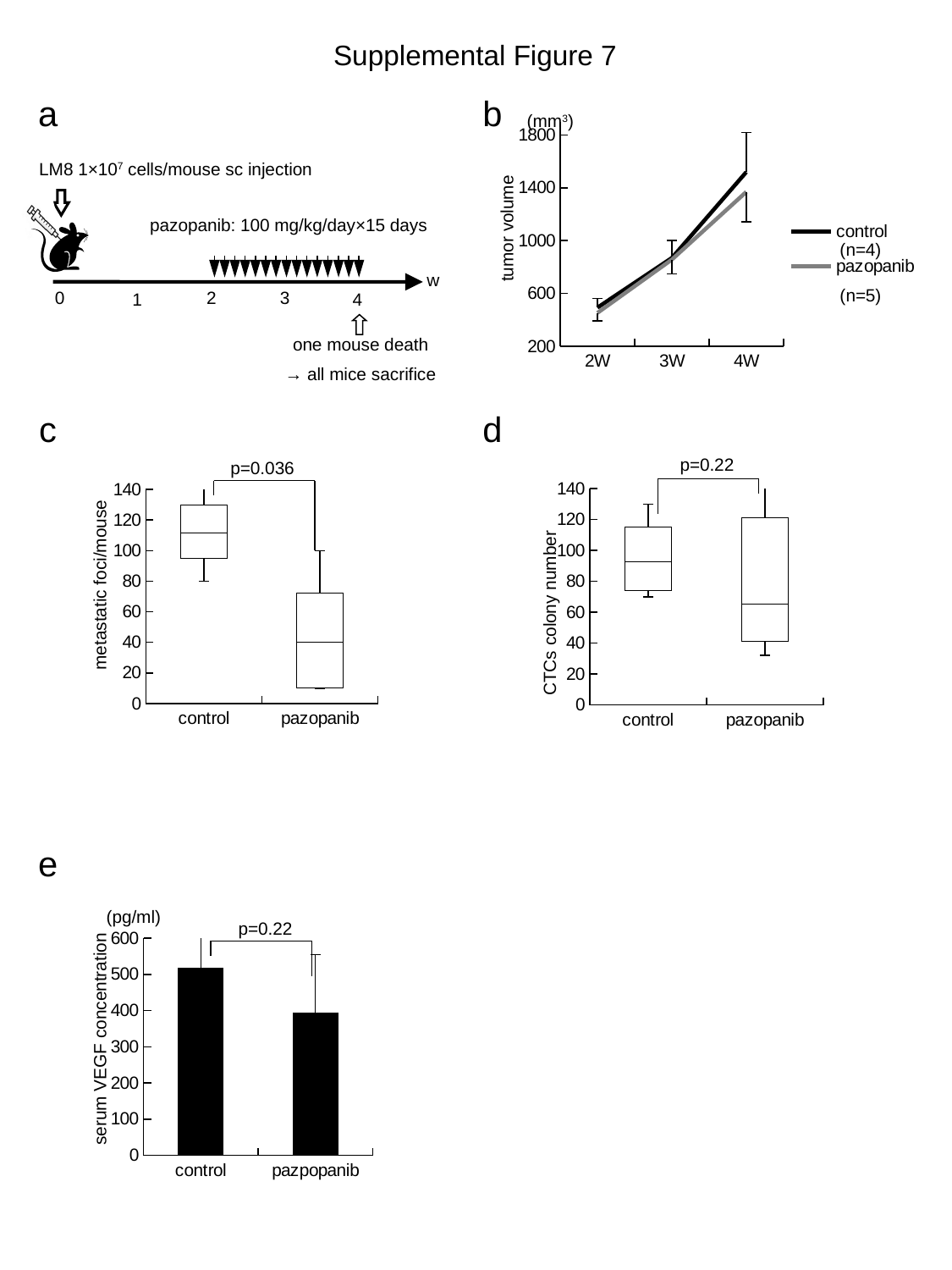

Supplemental Figure 7
a
b
(mm3)
### Chart
| Category | control | pazopanib |
|---|---|---|
| 2W | 493.0 | 452.9874999999994 |
| 3W | 868.9531249999987 | 856.4 |
| 4W | 1518.140625 | 1368.525 |tumor volume
LM8 1×10^7^ cells/mouse sc injection
pazopanib: 100 mg/kg/day×15 days
(n=4)
w
(n=5)
0
2
3
1
4
one mouse death
→ all mice sacrifice
c
d
### Chart
| Category | | | |
|---|---|---|---|
| control | 95.0 | 16.5 | 18.25 |
| pazopanib | 10.0 | 30.0 | 32.0 |metastatic foci/mouse
p=0.036
p=0.22
### Chart
| Category | | | |
|---|---|---|---|
| control | 73.75 | 18.75 | 22.5 |
| pazopanib | 41.0 | 24.0 | 56.0 |CTCs colony number
e
(pg/ml)
### Chart
| Category | |
|---|---|
| control | 519.0 |
| pazpopanib | 394.6 |serum VEGF concentration
p=0.22
