# Supplementary figures and images for: Dynamic analysis of lung metastasis by mouse osteosarcoma LM8: VEGF is a candidate for anti-metastasis therapy
Source: Clin Exp Metastasis. 2012 Oct 18;30(4):369–79. doi: 10.1007/s10585-012-9543-8 (PMC3616224; doi:10.1007/s10585-012-9543-8)

## Slide 1
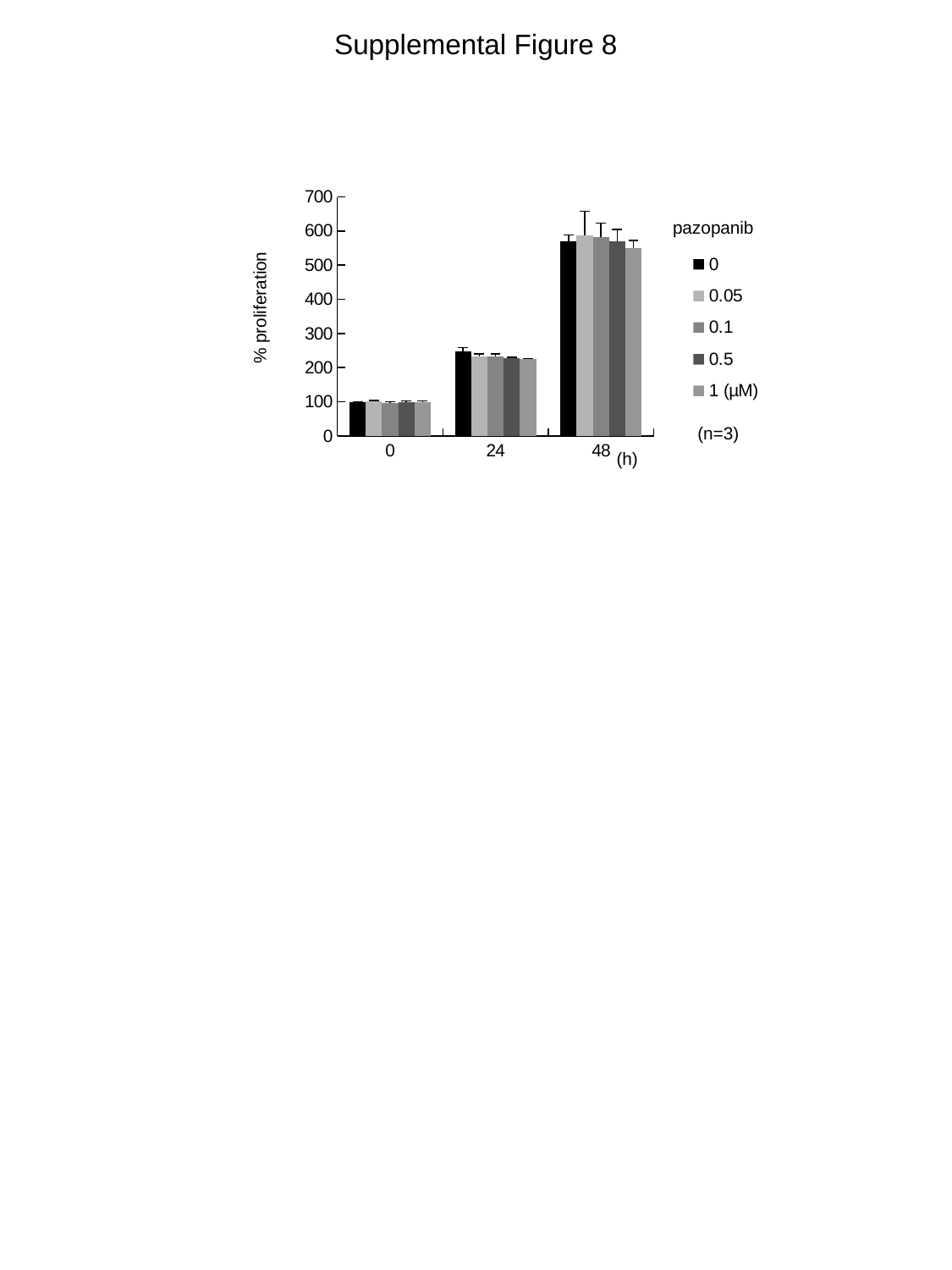

Supplemental Figure 8
### Chart
| Category | 0 | 0.05 | 0.1 | 0.5 | 1 (µM) |
|---|---|---|---|---|---|
| 0 | 100.0 | 100.17967263160374 | 97.15268580003314 | 98.09026847921956 | 98.96565279885131 |
| 24 | 246.76968788287255 | 233.7610161645463 | 233.68038866142427 | 226.7544861430694 | 224.44278044635618 |
| 48 | 571.1732948809245 | 587.0903158190816 | 583.6878351872476 | 571.100730128119 | 551.0855283883104 |pazopanib
% proliferation
(n=3)
(h)

Supplement: Supplementary file 8 — Supplementary material 8 (PPTX 36 kb) [file 10585_2012_9543_MOESM8_ESM.pptx]
